# Unraveling the Secrets of Early-Maturity and Short-Duration Bread Wheat in Unpredictable Environments

**DOI:** 10.3390/plants13202855

**Published:** 2024-10-12

**Authors:** Charan Singh, Sapna Yadav, Vikrant Khare, Vikas Gupta, Umesh R. Kamble, Om P. Gupta, Ravindra Kumar, Pawan Saini, Rakesh K. Bairwa, Rinki Khobra, Sonia Sheoran, Satish Kumar, Ankita K. Kurhade, Chandra N. Mishra, Arun Gupta, Bhudeva S. Tyagi, Om P. Ahlawat, Gyanendra Singh, Ratan Tiwari

**Affiliations:** 1 ICAR—Indian Institute of Wheat and Barley Research, Karnal 132001, India; 2 Nuclear Agriculture and Biotechnology Division, Bhabha Atomic Research Centre, Mumbai 400085, India; 3 Central Sericultural Research and Training Institute, Pampore 192121, India

**Keywords:** early maturity, bread wheat, breeding, QTL, wheat quality

## Abstract

In response to the escalating challenges posed by unpredictable environmental conditions, the pursuit of early maturation in bread wheat has emerged as a paramount research endeavor. This comprehensive review delves into the multifaceted landscape of strategies and implications surrounding the unlocking of early maturation in bread wheat varieties. Drawing upon a synthesis of cutting-edge research in genetics, physiology, and environmental science, this review elucidates the intricate mechanisms underlying early maturation and its potential ramifications for wheat cultivation in dynamic environments. By meticulously analyzing the genetic determinants, physiological processes, and environmental interactions shaping early maturation, this review offers valuable insights into the complexities of this trait and its relevance in contemporary wheat breeding programs. Furthermore, this review critically evaluates the trade-offs inherent in pursuing early maturation, navigating the delicate balance between accelerated development and optimal yield potential. Through a meticulous examination of both challenges and opportunities, this review provides a comprehensive framework for researchers, breeders, and agricultural stakeholders to advance our understanding and utilization of early maturation in bread wheat cultivars, ultimately fostering resilience and sustainability in wheat production systems worldwide.

## 1. Introduction

Over 10,000 years ago, in the Fertile Crescent of the Near East, wheat was domesticated, becoming one of the earliest and most extensively cultivated cereal crops. It is among approximately 300,000 commonly cultivated edible plant species [1], domesticated alongside rice and maize [2]. Together, wheat, rice, and maize contribute nearly half of the world’s caloric intake and two-fifths of its protein consumption. Providing one-fifth of the global calories and protein [3,4], wheat plays a vital role in ensuring global food and nutrition security. Cultivated on approximately 218.5 million hectares worldwide, the majority of wheat-growing regions lie in the northern hemisphere. The distribution of wheat in these regions depends on various species, varieties, and their adaptability to diverse environments [5]. The average annual cereal yield per hectare stands at 3.4 tons [6]. Extreme weather conditions, notably high temperatures and limited water supply, significantly impact wheat production [7]. Heat stress affects crucial stages like flowering and kernel filling, hindering sustainable high yield output. Elevated seasonal temperatures during grain filling lead to 15–35% yield reductions in Africa and Asia, and 25 to 35% in the Middle East [8,9]. Moreover, global warming escalates disease and parasite infestations, exacerbating production challenges [10]. These factors, combined with population growth, urbanization-induced agricultural land decline, and resource scarcity due to climate change, threaten wheat production sustainability, leading to severe economic and social repercussions [10,11]. To meet the increasing demand driven by population growth, wheat production must annually increase by 2% [12]. Breeding efforts focus on high-yielding, early-maturing cultivars, crucial for intensive cropping systems and avoiding adverse conditions during grain filling [13]. Evaluating genetic parameters for agronomic and physiological traits is essential for identifying superior progenitors and hybrids, particularly stress-tolerant varieties. The traits of interest include long coleoptiles for deeper sowing, reduced tillering, and early vigor, enhancing plant vitality and water-use efficiency [14]. Early canopy closure, quantified by NDVI, enhances crop competitiveness and correlates with leaf width and embryo size, impacting leaf area and biomass [15,16,17]. These genetic traits interact with environmental factors and soil conditions, affecting wheat performance and yield [8,18,19]. This review discusses the meticulous selection of progenitors and germplasm characterization for wheat breeding. Wheat’s growth phases, influenced by the vernalization requirement, photoperiod response, and earliness genes, are crucial for its adaptation and yield potential in various environments [20,21]. Breeding methods, including pure-line selection, MAS, QTL mapping, and speed breeding, aim to develop early-maturing wheat genotypes. Mutations in genes regulating GA and ABA biosynthesis lead to shorter plant stature and accelerated early embryo development, fostering early maturation and optimizing nutrient uptake and distribution during rapid growth and grain filling [22]. Early maturation is a vital trait in wheat cultivars, particularly in regions prone to post-heading temperature stress. It allows wheat to complete grain filling under favorable conditions, avoiding late-season heat stress that can diminish grain production and quality. Cultivars with earliness, faster grain-filling rates, and prolonged grain-filling durations are advantageous by allowing more time for dry matter assimilation during the wheat growing season. Despite its importance, there is limited understanding of how early-maturing wheat genotypes affect nutrient assimilation and transport, necessitating further research. Elucidating the genetic, molecular, and biochemical mechanisms underlying reduced photoperiod sensitivity is essential for developing early-maturing wheat cultivars, and enhancing global food security and agricultural sustainability. This review, “unraveling the secrets of early maturity and short duration bread wheat in unpredictable environments”, explores the mechanisms behind early maturity and short duration in bread wheat, particularly in the context of unpredictable environmental conditions. It seeks to uncover the genetic, physiological, and agronomic factors that contribute to these traits, with the goal of improving wheat varieties to ensure better yields and stability in diverse and challenging environments.

## 2. Early-Maturity and Short-Duration Bread Wheat

Heat stress affects wheat differently across various phenological stages, with a more pronounced impact during reproductive phases due to its direct effect on grain number and dry weight [23]. In South Asia, regions like the North Eastern Plain Zone (NEPZ), North Western Plain Zone (NWPZ), Central Zone (CZ), and Peninsular Zone (PZ), as well as areas in Bangladesh and Pakistan, are particularly prone to heat stress [24,25]. Two types of high-temperature stress occur, namely terminal stress during grain-filling stages and persistent stress throughout the growing season. Early-maturing wheat lines adapt by completing seed setting and grain filling under favorable conditions, escaping late heat stress and optimizing resource utilization during ongoing stress [26,27]. Tolerance to heat stress is associated with genotypes possessing stable photosynthesis and high stem reserve capacity [28,29]. Breeding efforts should prioritize the development of wheat lines suited for warm climates, particularly high-yielding, early-maturing varieties equipped to withstand both temporary and ongoing high-temperature stress conditions.

## 3. Characteristics of Early-Maturing Varieties

### 3.1. Drought Escape (DE)

This is one of the strategies adopted by plants as and when water is scarce; the plant is able to finish its life cycle quickly during a short period of favorable growth conditions. Advanced cell expansion and cell division, as well as a high metabolic rate, are linked to this. Stomata opening and increased gas exchange enable faster photosynthesis and photo-respiration even in conditions of limited water availability, resulting in rapid plant development [30]. Despite the traditional classification of DE in plant ecology as a trait of “ephemeral native plants”, significant cereals like wheat and barley can exhibit a mechanism resembling DE known as “earliness” or “early flowering” [31].

### 3.2. Time to Initiation of Flowering

Early flowering or heading in wheat has been recommended as a useful technique for wheat breeding for terminal high-temperature stress, because it is an important escape mechanism under the late incidence of high-temperature stress [24,25].

### 3.3. Cooler Canopy Temperatures

Cooler canopy temperatures (CTs), allow plants to retain physiological activities at high temperatures and have been linked to grain yield under warm irrigated conditions [32]. Under water-scarce conditions, canopy temperature (CT) may be a good indicator of a plant’s capacity to extract water [33]. Maintaining high leaf chlorophyll content is also seen as a desirable characteristic because it shows that the photosynthetic system is not significantly photo-inhibited at high temperatures [34].

### 3.4. Grain Filling Duration

The key factors affecting wheat maturity are the time to anthesis and the ensuing interval from anthesis to physiological maturity (grain-filling period). Both the length and the duration of grain filling are impacted by high temperatures. According to some studies, high temperatures cause grain-filling rates to increase and grain-filling times to shorten [35,36]. The rate of grain growth appears to rely on whether the number of grains per spike is decreased as temperature rises. Spikelets slow down grain growth in spikes where the number of grains is less impacted by high temperatures. The shorter grain-filling period should therefore be compensated by an increase in grain filling rate, but this does not happen at temperatures above 30 °C [37]. However, according to other investigations, longer grain-filling times under heat stress were not offset by higher grain-filling rates [38,39]. Furthermore, showed that heat stress reduced both the duration and rate of grain growth in wheat.

### 3.5. Grain Number and Size

Increased temperatures shorten the interval between anthesis and physiological maturity, which is linked to decreased grain weight [40]. For every degree Celsius over 15 to 20 degrees, grain weight can be reduced by 1.5 mg per day [41]. Rising temperatures (day/night temperatures rose from up to 31/20 °C) can cause grain shrinking through detrimental ultra-structural changes in the aleurone layer and endosperm cells in wheat seeds [42].

### 3.6. Thousand-Kernel Weight (TKW)

Various studies have reported that high temperature during grain filling leads to a reduction in thousand-kernel weight [43]. However, an experiment carried out by [13] showed that wheat lines with high thousand-kernel weight also maintained higher TKW across different locations. It was also observed that TKW does not have a significant association with earliness; however, early-maturing entries were able to maintain TKW even under stressful conditions.

### 3.7. Stay Green Trait

**Figure 1:**
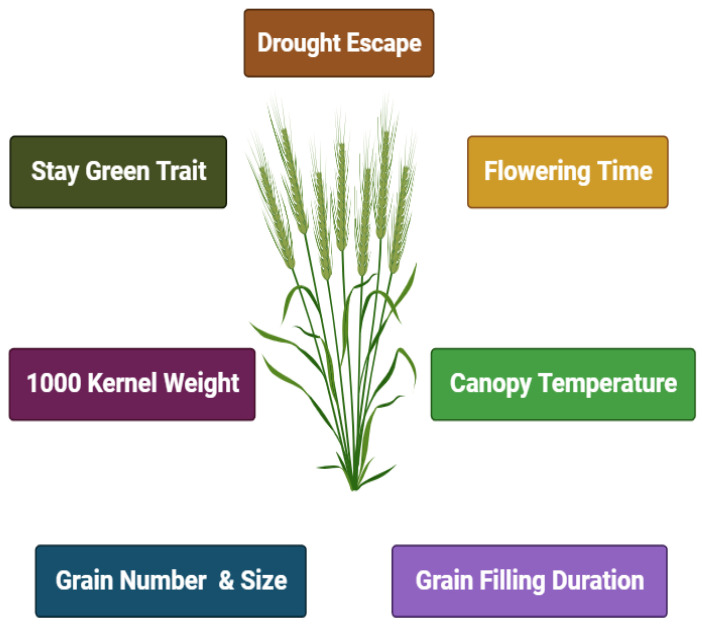
Characteristics of early-maturing wheat genotypes.

In response to heat stress, leaf senescence begins early, especially during the post-flowering stages of grain filling. As a result, maintaining leaf chlorophyll and photosynthetic ability, known as “stay-green”, is considered to be a sign of heat tolerance. Stay-green genotypes should be better able to maintain grain filling under high temperatures, because the loss of chlorophyll is linked to a reduced uptake of current carbon into grains [36] (Figure 1).

## 4. Biochemical Perspectives of Early-Maturing Wheat Genotypes and Their Effects on Quality Attributes

Early-maturing wheat genotypes offer benefits such as reduced susceptibility to environmental stress and enhanced adaptability to diverse conditions. The development of these genotypes involves complex biochemical processes governing wheat growth and maturation. Despite the urgency imposed by rising global temperatures, there is a lack of significant research dissecting the biochemical mechanisms underlying early maturity in wheat. However, pathways associated with photoperiod sensitivity, hormonal regulation, and carbohydrate metabolism are believed to play crucial roles in wheat’s early maturation.

### 4.1. Reduced Photoperiod Sensitivity in Early-Maturing Wheat

Photoperiod sensitivity denotes a plant’s responsiveness to day length, particularly its influence on flowering and subsequent maturation stages. While day length serves as a pivotal cue for these processes in many plants, such as wheat, early-maturing wheat genotypes necessitate reduced photoperiod sensitivity to bloom and mature under shorter day lengths [44]. This reduced sensitivity forms a crucial component of the biochemical pathway leading to early maturity in wheat, enabling varieties to flower and mature irrespective of day length, thus fostering multiple cropping seasons and bolstering resilience to environmental challenges [45]. At the core of this reduced sensitivity in wheat lies the *Photoperiod 1* (*Ppd-1*) gene, encoding a transcription factor that modulates the plant’s response to day length. *Ppd-1* exhibits diverse allelic variants, some of which confer reduced photoperiod sensitivity [46]. Notably, studies by [45,47] underscored the significance of specific mutations within *Ppd-1*, revealing their association with reduced photoperiod sensitivity and early maturity in wheat. This reduced sensitivity significantly impacts wheat cultivation, enabling its adaptation to varying day lengths and facilitating multiple cropping seasons, ultimately enhancing yields. Furthermore, early-maturing wheat genotypes display heightened resilience to adverse weather conditions, completing their life cycle and yielding harvests before unfavorable conditions escalate. By maturing early, these wheat varieties optimize resource allocation, enhancing grain development and quality, thus augmenting crop yield. In essence, reduced photoperiod sensitivity is a cornerstone of the biochemical pathway governing early maturity in wheat. The elucidation of the detailed genetic, molecular, and biochemical mechanisms underlying this trait is indispensable for the development of early-maturing wheat genotypes, thereby contributing to global food security and sustainable agriculture [48].

### 4.2. Hormonal Regulation in Early-Maturing Wheat

Recent research has shed light on the crucial role of hormonal regulation in governing early maturity across various crops, including wheat. Specifically, gibberellins (GAs) emerge as pivotal phytohormones driving this process in wheat genotypes. By orchestrating plant height reduction, GAs facilitate early maturation, a desirable trait in wheat breeding for lodging prevention and enhanced grain development [22]. Mutations in GA biosynthesis genes, such as *Reduced Height* (*Rht*) genes, lead to decreased stature and expedited maturation. Additionally, GAs promote flowering in wheat by modulating metabolic and signaling pathways, facilitating earlier reproductive development in early-maturing genotypes [44].

Recent research has illuminated the role of abscisic acid (ABA) in wheat’s early maturity, contrasting its traditional association with stress responses and dormancy induction. The authors of [49] found that non-dormant wheat mutants exhibited ABA insensitivity and reduced ABA levels during seed development, suggesting a contribution to early maturation, while the authors of [23] showed ABA’s role in regulating gene expression in wheat embryos, potentially influencing early maturation processes. Ref. [50] observed a significant ABA increase during wheat grain development, linking it to maturation regulation. Understanding these hormonal mechanisms is pivotal for developing early-maturing wheat varieties, crucial for global food security amidst changing environmental conditions. Research into hormonal regulation promises resilient and adaptable wheat varieties, supporting sustainable agriculture and food security efforts.

### 4.3. Carbohydrate Metabolism

The role of carbohydrate metabolism in wheat’s early maturity is significant. Ref. [51] examined the impact of magnesium sulfate foliar application on wheat grain filling, revealing positive effects on photosynthetic characteristics and carbohydrate metabolism. This enhancement may accelerate grain filling and indirectly contribute to early maturity in wheat, though further investigation is needed to ascertain its exact impact. Ref. [52] explored the spatial distribution of proteins and metabolites during wheat grain development, offering insights into regulatory processes that could enhance early maturity and grain-filling efficiency, potentially leading to improved yield and maturation characteristics. Ref. [53] demonstrated that high nighttime temperatures disrupt carbon balance and metabolic processes in winter wheat, potentially leading to early maturity or impaired grain development, highlighting the significance of temperature fluctuations. Ref. [54] found that low temperatures during the booting stage affect sucrose metabolism and hormone levels in wheat spikelets, potentially impacting reproductive processes and early maturity. Certain wheat genotypes or environmental conditions may influence photosynthesis and associated biochemical processes, affecting maturity timing due to variations in carbon assimilation, nutrient uptake, or other physiological factors.

### 4.4. Nutrient Uptake and Transport

The efficient acquisition and distribution of essential nutrients during the reproductive stage are critical for the growth and development of crop plants, particularly early-maturing wheat genotypes, facilitating rapid growth, development, and grain filling. Despite the importance of nutrient uptake and transport, there has been limited progress in understanding their effects on early-maturing wheat genotypes, necessitating urgent investigation. We hypothesize that early-maturing wheat genotypes often exhibit a heightened expression of the genes responsible for nutrient uptake, including those for nitrogen (N), phosphorus (P), and iron (Fe), potentially leading to improved nutrient availability during critical growth stages, thereby promoting rapid maturation and grain development. Additionally, the roots of early-maturing wheat genotypes are equipped with efficient nutrient absorption systems, which may include specialized transporters and beneficial associations, such as mycorrhizal symbiosis, enhancing the root’s ability to absorb essential nutrients from the soil and ensuring an adequate supply of elements required for wheat growth and development. Moreover, nutrient uptake and absorption efficiency are often linked to the timing of nutrient availability in the soil, suggesting that early-maturing wheat genotypes may possess mechanisms allowing them to acquire nutrients when most needed for reproductive growth, thus accelerating the maturation process. Understanding these mechanisms is crucial for the development of wheat varieties with early maturation characteristics, and further research into nutrient uptake and transport will provide valuable insights for improving nutrient use efficiency and enhancing the early maturity of wheat.

### 4.5. Wheat Quality Attributes and Early Maturity

Protein content is a critical quality parameter in wheat, profoundly impacting flour and dough functionality. Early-maturing wheat varieties often show reduced protein content due to their shorter vegetative growth phase, limiting nitrogen uptake [55]. This can lead to weaker dough with lower elasticity and loaf volume, affecting bread quality, and influencing pasta and noodle texture and cooking properties. However, quality implications vary based on end-use and other wheat attributes. Variations in grain size and kernel hardness are evident in early-maturing wheat, crucial for milling efficiency and end-product quality. Smaller grain size may reduce flour yield and impact flour composition, while kernel hardness affects suitability for specific products [56]. Starch quality is central to noodle production, influenced by amylose and amylopectin content. Early-maturing wheat may exhibit altered starch quality, affecting noodle texture and cooking properties [57]. Dough rheology, including strength, extensibility, and elasticity, is vital for end-product suitability. Early-maturing wheat genotypes may vary in dough rheological properties, affecting product quality.

## 5. Conventional Breeding Approaches for Enhancing Earliness and Short Duration in Bread Wheat

During the grain-filling stage, wheat yield and nutritional quality are significantly influenced by edapho-climatic factors. Environmental factors such as temperature, humidity, and water availability strongly influence wheat spikelet flowering [58]. Earliness, an important trait, aids crops in evading environmental stresses like drought, heat, and cold, thereby enhancing productivity [59]. Earliness is not merely a single trait but rather an outcome of various flowering mechanisms, including photoperiod dependency, cold response, hormonal regulation, and autonomous pathways [60,61,62]. Genes like *FLOWERING LOCUS (FLC)*, *FLOWERING LOCUS T (FT)*, *CONSTANS (CO)*, *GIGANTEA (GI)*, *APETLLA1 (AP1)*, *LEAFY (LFY)*, among others, play pivotal roles in regulating flowering in angiosperms [62]. Flowering time is influenced by factors such as photoperiod sensitivity, vernalization requirement, and narrow-sense earliness, controlled by multiple and homoallelic genes [54,58,63,64,65]. The complex interaction of these factors, combined with wheat’s ploidy level, complicates genetic analysis and breeding efforts for early-maturing wheat cultivars. Nevertheless, initiatives have been undertaken to breed for earliness in wheat, resulting in significant progress in developing early-maturing cultivars.

Several researchers have conducted genetic analyses of earliness in wheat through the development of segregating populations [58,63,65,66]. Understanding the genetic patterns of earliness is crucial for developing wheat varieties with high grain productivity, protein content, and resistance to biotic and abiotic stressors. Ref. [58] assessed narrow-sense earliness in four segregating populations, observing a range of maturity from 29.8 to 51.2 days, indicating a polygenic nature with broad-sense heritability ranging from 0.90 to 0.99. Ref. [65] investigated variation for earliness in the Triticum and Aegilops species, identifying mutants with differing photoperiod responses, and suggesting control by recessive genes. Ref. [67] analyzed early generations, determining additive and dominance variance for all traits, with significant additive gene action observed for earliness and yield traits [68]. Combining ability analysis by [69,70] using half-diallel analysis revealed significant general combining ability (GCA) and specific combining ability (SCA) for earliness and other traits in segregating populations.

### 5.1. Pure-Line Selection

Selection within a single population is a fundamental method in plant breeding. Pure lines, resulting from the self-pollination of a single homozygous parent, are key outcomes of this method, allowing for the creation of progenies with desired traits such as early onset and short duration. Genetic correlations between leaf area and other traits like plant biomass, leaf breadth, length, specific leaf area, and coleoptile tiller frequency have been observed, suggesting potential indicators for early vigor in segregating wheat populations [71]. Selection for leaf breadth, correlated with leaf area, offers a rapid and non-destructive screening method for early vigor. Superior second-generation progenies undergo initial yield trials, followed by multi-location trials to assess performance across environments. After several years of trials, superior genotypes are released as new varieties, a process taking about six to seven years. However, phenotypic variances in pure lines are primarily influenced by environmental factors, limiting the effectiveness of selection due to low heritability [72]. In cotton, ref. [73] observed variations in days to 50% flowering, ranging from 49 to 103 days, and employed a pedigree-based approach for early flowering selection. While initial selection showed significant gains, subsequent generations demonstrated less reduction in flowering time. The developed lines offer potential for new cropping systems, such as pigeon pea-wheat, expanding production to nontraditional areas and providing farmers with wider planting time flexibility.

### 5.2. Mass Selection

Mass selection is a method employed to enhance the productivity of the base population by increasing the occurrence of desirable traits. The selection of plants based on phenotypic traits, such as earliness and short duration, is crucial in developing early vigor in wheat, which can improve water-use efficiency and production [71]. The presence and type of genetic variation for early vigor and its components play a critical role in optimizing selection efficiency in breeding programs. This method is particularly effective when the chosen traits have a high heritability [74], with additive genes contributing significantly to variance, thereby enhancing the efficiency of mass selection [75]. Mass selection can be conducted in two ways, namely single parental selection, where one gamete type is controlled, or bi-parental selection, where both male and female gametes are controlled. Bi-parental selection is more effective, as it allows for the selection of both parents [76]. The process begins by selecting a landrace based on key traits such as height, disease resistance, and maturation timing. The chosen individual is then grown in the field and harvested at maturity. Harvested seeds are mixed and utilized for subsequent generations. In the second year, crops are grown in large quantities using mixed seeds and compared to a control (check) variety in a preliminary yield trial. Over the next 3 to 4 years, varieties are tested at multiple locations alongside a check variety. After seven years of selection, the chosen plant is released as a new variety, and seeds are distributed to farmers.

### 5.3. Backcross Breeding

In this method, donor parents possessing desired traits for earliness and short duration are crossed with recurrent parents, which receive the selected genes, resulting in hybrid progeny (F1). The progeny of F1 is then selected for the desired traits of earliness. Selected F1 progenies are grown, and backcrossing with the recurrent parent produces backcross hybrids (BC1). BC1 generations are further selected for desired traits and cultivated in different fields. Subsequently, BC1 individuals are re-crossed with recurrent parents, and this process is repeated until the sixth backcross generation (BC6) is obtained. The resulting BC6 generation is then raised for seed production. To ensure the efficacy of the breeding process, multiple yield experiments are conducted at various sites, including a recurrent parent as a check variety. It is imperative that the hybrid closely resembles the recurrent parent, typically up to 98% [77]. In backcross breeding, inconsistent gene contributions from the two progenitor lineages are common, with fewer genes being contributed by the donor parent compared to the recurrent parent [78].

## 6. Modern Breeding Approaches

Traditional breeding faces challenges in phenotypic selection due to masking effects, making it difficult to select quantitative traits governed by multiple alleles, as their phenotypic expression varies. To address these challenges, breeding operations increasingly integrate other branches of biology to enhance success and efficiency.

### 6.1. Genomic Selection

Genomic selection (GS) has been widely utilized in agriculture over the last decade to develop complex polygenic traits for climate resistance. It employs prediction models, estimating the influence of all markers on a desired phenotype. Unlike traditional breeding, GS accelerates grain production, surpassing the efficacy of marker-aided selection by leveraging genome-wide markers to estimate breeding values based on quantitative gene loci [79]. The prediction of breeding values is achieved through penalized regression and Bayesian approaches, with the quality of predictions evaluated by assessing the connection between estimated and actual breeding values [80]. The assessment of breeding values utilizes both training and breeding populations, where the phenotype and genotype training population are used to predict breeding values in the breeding population [81]. Importantly, genomic selection effectively identifies small-effect genes with multiple quantitative trait loci (QTLs), which play a significant role in controlling economically and agronomically important traits in plant breeding [82].

### 6.2. Marker-Assisted Selection

Marker-assisted selection (MAS) utilizes DNA markers to pinpoint genomic regions in plants exhibiting desirable traits [83]. Its efficiency in breeding operations is enhanced by the use of multiple DNA markers. Among the widely employed markers in plant breeding are Amplified Fragment Length Polymorphism (AFLP), simple sequence repeats (SSRs), also known as microsatellites, and Single Nucleotide Polymorphism (SNP), particularly for traits like earliness and short duration in wheat crops. SSRs are particularly favored due to their simplicity and cost-effectiveness [84]. One of the key advantages of DNA markers is their independence from plant phenotype and resistance to the influence of growing environment or heredity [27]. MAS excels in selecting traits related to late reproductive stages, such as grain yields, flower colors, and seed attributes, with DNA markers capable of identifying genetic traits early in plant growth [85]. It proves especially effective in selecting less heritable, expensive to execute, and complex traits. However, its efficacy is limited to selecting only a few quantitative traits that exhibit significant variation among populations.

### 6.3. QTL Mapping

Over the past two decades, advancements in molecular markers and genome sequencing in wheat have empowered plant breeders to locate genomic regions linked to specific traits. The adoption of genomic breeding has significantly advanced wheat production worldwide. However, escalating challenges posed by population growth and climate change have emerged as top priorities for global food security, compelling wheat communities to boost productivity through cutting-edge technologies. Earliness stands out as a crucial trait in addressing the challenge of climate change by modifying genotypes to withstand environmental pressures during the growing season, leading to the development of early-maturing varieties with enhanced productivity. In pursuit of this goal, the identification, mapping, and introgression of genomic regions/QTLs associated with earliness and its related traits have been emphasized [86]. Utilizing diverse diversity panels and introgression lines, numerous studies [18,87,88] have successfully identified QTLs/genes in wheat.

Mapping QTLs associated with physiological traits such as earliness and short duration is pivotal for marker-assisted breeding in wheat, as highlighted by studies conducted by [89,90,91,92]. Table 1 lists the QTLs linked to phenological and physiological traits relevant to earliness. Enhancing wheat production can be achieved by transferring these discovered QTLs to either new or well-adapted cultivars [93]. Various genomic regions have been identified for phenological and yield-related parameters like grain yield, thousand-kernel weight, biomass, and days to heading [94], suggesting a set of linked and/or co-located QTLs influencing these traits. According to [94], identifying QTLs associated with days to heading and thousand-grain weight implies that early maturity could extend grain-filling duration post anthesis, resulting in a larger grain size and an increased grain output. Quantitative trait loci governing both flag leaf qualities and traits linked to yield have been uncovered on chromosomes 1B, 2D, 4A, 4D, 4B, 5A, 5B, 6B, 6D, and 7D in wheat, as reported by [95,96]. Thus, expediting the breeding process to enhance genetic gains in wheat grain yield necessitates a thorough analysis of genomic regions influencing physiological features. Additionally, it is imperative to develop markers associated with these traits to strategically advance breeding objectives.

### 6.4. Field Phenomics

Accelerated plant breeding for climate resilience relies significantly on precise and efficient field-level phenotyping, enabling the effective evaluation and selection of superior breeding lines across diverse environments [108]. Over the past decade, the adoption of novel sensors such as unmanned air vehicles (UAVs), high-resolution imagery, and new platforms has substantially increased the collection of phenotypic data, advancing the field of phenomics [109,110]. High-throughput phenotyping (HTP) plays a pivotal role in efficiently screening plant architectural traits and the early identification of desirable genotypes. It facilitates precise, automated, and replicable measurements for a wide range of agronomic traits, including seedling vigor, flowering time, flower counts, biomass and grain yield, height, leaf erectness, and canopy structure. Furthermore, it enables the assessment of physiological traits, such as photosynthesis, disease resistance, and stress tolerance. Various HTP methods including RGB imaging, 3-D scanning, thermal and hyper-spectral sensing, and fluorescence imaging have proven effective in identifying, quantifying, and monitoring plant diseases [111]. When combined with high-throughput phenotyping facilities, Genome-Wide Association Studies (GWAS) allow for the utilization of phenomics as a novel tool in plant genetics and genomic characterization, thus enhancing crop breeding efficiency in response to climate change [112]. Moreover, the increasing utilization of Deep Learning (DL) techniques facilitates the analysis and interpretation of large-scale phenomics data, particularly in plant image analysis and environmental stress phenotyping [113].

### 6.5. Speed Breeding

**Figure 2:**
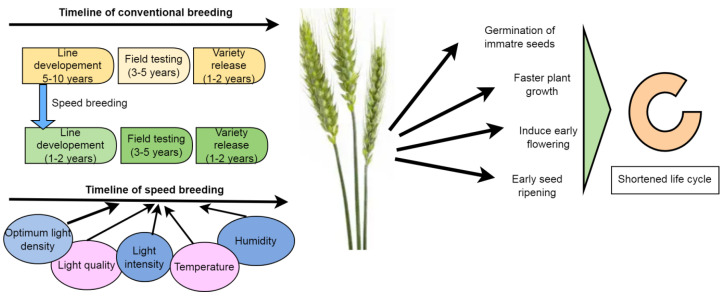
The procedure of speed breeding in variety release through shortening the life cycle of wheat.

Speed breeding has emerged as a potent tool in addressing global food security and mitigating the impacts of climate change. This technique accelerates plant growth by inducing early seed germination, flowering, and seed ripening, optimizing crop photoperiod, light density, intensity, temperature, and humidity (Figure 2). By employing speed breeding, the time taken for variety release is reduced by 4–8 years compared to conventional breeding methods, facilitating faster line development (Figure 2). It expedites trait selection and the development of climate-resilient crops. Moreover, speed breeding enables the discovery of genetic variety and valuable traits that enhance agricultural performance [114]. Other approaches such as shuttle breeding, the doubled haploid technique, and off-season crops have also been employed in the past to accelerate the breeding cycle. For instance, the wheat variety Blaise (University of Queensland) was developed using speed breeding, exhibiting early maturity, high yield potential, and resistance to foliar diseases [115]. Controlled speed-breeding facilities have been established in India for crops like pigeon pea, chickpea, and pearl millet at ICRISAT, Hyderabad, and for rice at ISARC, Varanasi. Additionally, controlled speed-breeding facilities for wheat are under development at PAU-Ludhiana, ICAR-IARI-New Delhi, and ICAR-IIPR, Kanpur [116]. Combining speed breeding with modern breeding techniques such as plant phenotyping, marker-assisted selection, marker-assisted backcrossing, genomic selection, genome editing, and express editing can pave the way for the development of climate-smart crop varieties. Harnessing speed breeding alongside modern breeding tools holds the potential to usher in a second green revolution capable of feeding billions of people [116].

## 7. Genetic Basis of Earliness in Wheat

**Figure 3:**
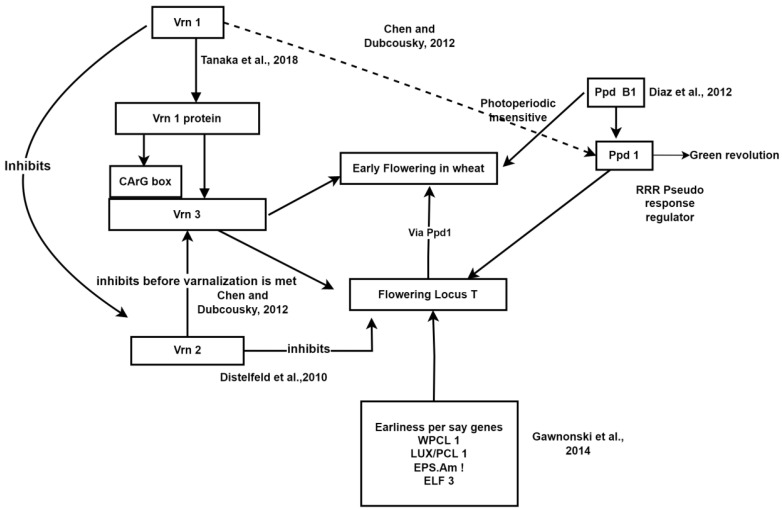
The proposed mechanisms by [44,126,130,131,141] illustrate the molecular basis of the genetic control of earliness.

The genetic analysis of economic traits is crucial for developing plant varieties suited to varying agro-climatic conditions and designing effective breeding programs. Understanding the number and type of genes involved in controlling specific traits is particularly helpful in this regard. In wheat, the variability for earliness is divided into three components: photoperiod sensitivity (PS), vernalization requirement (VR), and intrinsic earliness (IE) [103]. These components influence various developmental stages such as kernel number per spikelet, rate of kernel growth, and duration of fill, all of which significantly contribute to wheat productivity [117]. Numerous studies have explored the genetic analysis of earliness using classical Mendelian genetics and cytological methods. For instance, refs. [64,118] investigated the *Ppd* gene sets on group 2 chromosomes, while ref. [119] focused on the *Vrn* gene sets on chromosome group 5. Developing early-maturing wheat genotypes not only enhances productivity but also extends wheat cultivation from colder to warmer areas during the growing season [66]. Before the green revolution in India, tall wheat genotypes predominated, which were photoperiod-insensitive and late in maturity. However, the introduction of dwarf wheat genotypes like Lerma Rojo and Sonora 64, which carry photoperiod-insensitive genes (*Ppd-1* and *Ppd-2*), revolutionized wheat cultivation in India. The varied influence of *Ppd* genes on wheat cultivation has enabled its adaptation to diverse agro-climatic conditions [120]. Additionally, the *Ppd-1* gene plays a crucial role in balancing the transition between the vegetative and reproductive phases [66]. Alongside *Ppd* genes, allelic diversity in vernalization requirement genes (*Vrn*) determines the duration of the vegetation period and can convert winter wheat into spring wheat [63]. To date, five *Vrn* genes (*Vrn-A1*, *Vrn-B1*, *Vrn-D1*, *Vrn-B3* and *Vrn-D4*) [121,122,123,124,125] and three *Ppd* genes (*Ppd-B1*, *Ppd-D1* and *Ppd-A1*) [45,126] have been identified in bread wheat. The growth and developmental stages of wheat, encompassing tillering, stem elongation, ear emergence, anthesis, and ripening, are intricately regulated by photoperiod response, vernalization requirement, and genes associated with earliness [127]. Vernalization requirement and photoperiod sensitivity play crucial roles in accelerating or delaying ear initiation to protect delicate floral primordia from high temperatures during adaptation [127], responding to specific day length and temperature cues [128]. The interaction of these genes for early flowering is illustrated in Figure 2. Four primary vernalization requirement genes, namely *Vrn1*, *Vrn2*, *Vrn3*, and *Vrn4*, have been identified in wheat, with the chromosomal location of interest situated on the long arm of the homoeologous group 5 chromosomes. *Vrn1* has been recognized as an orthologous counterpart of *APETALA1* (*AP1*), a MADS-box-like gene found in *Arabidopsis*, as reported by [122]. Notably, a significant loss in the *Vrn1* promoter or first intron reduces the vernalization requirement [129]. Positioned upstream of *Vrn3* in the flowering pathway, *Vrn1* directly interacts with the CArG box in the promoter region of *Vrn3*, as evidenced by [130]. The *Vrn2* locus comprises two tandem genes (*ZCCT1* and *ZCCT2*) encoding zinc finger proteins. *Vrn2* functions to repress *Vrn3* before the vernalization requirement, thereby preventing winter flowering [131]. Following sufficient low temperature exposure, *Vrn1* enhances leaf *Vrn3* expression by inhibiting *Vrn2* [131,132]. In wheat, *Ppd1*, the primary photoperiod-sensitivity gene, exhibits orthology with the Pseudo-Response Regulator (*PRR*) genes of *Arabidopsis thaliana* [133]. Possessing photoperiod insensitivity alleles, *Ppd1* is recognized as a “Green Revolution” gene, conferring adaptability to various temperate agricultural conditions. At the *Ppd-D1* locus, the presence of *Ppd-D1a*, featuring a 2089 bp deletion in the promoter region, induces photoperiod insensitivity and early flowering in common wheat [45,134,135]. Among the key genes controlling the photoperiod system in wheat are *Triticum aestivum GIGANTEA* (*TaGI*), *Wheat CO1* (*WCO1*), and *Triticum aestivum heading date 1* (*TaHd1*). *TaGI*, resembling the *Arabidopsis GIGANTEA* ortholog [136], along with *WCO1* and *TaHd1*, which are orthologs of *CONSTANS* in *Arabidopsis* [137,138], are pivotal in regulating wheat’s photoperiod response. While some researchers suggest that *Vrn* genes account for up to 75% of the variability in the vegetation period (earliness), *Ppd* genes are estimated to control approximately 20% [139]. In recent studies by [140,141], *WPCL1*, identified as an ortholog of *LUX ARRHYTHMO/PHYTOCLOK1* (*LUX/PCL1*) in *Arabidopsis*, was discovered. This gene, located on chromosome 3A, plays a crucial role in regulating circadian rhythms, as demonstrated by its deletion leading to impaired circadian rhythms. Additionally, ref. [142] identified *Eps-Am1* as a potential gene associated with earliness in einkorn wheat (*T. monococcum*). *Eps-Am1* is identified as an ortholog of the circadian clock regulator *EARLY FLOWERING3* (*ELF3*) found in *Arabidopsis*. Research into the molecular mechanisms governing the genetic regulation of earliness in wheat is gaining momentum [143]. However, there remains significant confusion regarding the phenotypic expressions of earliness, influenced by the interplay between alleles of the *Vrn* and *Ppd* genes. A comprehensive understanding of the genetic determinants controlling the timing of flowering is crucial for effectively influencing phenological developmental processes and enhancing the yield potential in wheat [144] (Figure 3).

## 8. Perspective of Genetic Modifications for Earliness in Wheat

In the current context, the trait of earliness holds significant importance, especially in regions facing reduced yields due to drought and extreme heat challenges. Breeding initiatives aimed at developing novel wheat genotypes characterized by early flowering and maturity are urgently needed to address these issues [7,17,145]. Understanding the genetic factors governing flowering timing is crucial for manipulating phenological development processes to enhance wheat yield potential [144]. Vernalization requirement and photoperiod-insensitive genes play critical roles in developing early-maturing wheat genotypes with high yield potential. Winter wheat relies on the vernalization requirement, or prolonged exposure to low temperatures, to induce flowering, while spring wheat exhibits lower vernalization requirement and can flower without cold exposure [82]. The response to cold exposure is regulated by vernalization requirement genes such as *VRN1*, *VRN2*, *VRN3*, and *VRN4* [146,147]. *VRN1*, encoding a MADS-box transcription factor, plays a pivotal role in cold response regulation [122]. During the vegetative phase, *VRN1* remains inactive but becomes active upon exposure to low temperatures, leading to increased expression. Up-regulated *VRN1* negatively affects *VRN2*, encoding zinc finger CCT domain proteins. *VRN2* acts as a negative regulator for *VRN3*. Cold exposure boosts *VRN1* expression, suppressing *VRN2* and consequently activating *VRN3*. *VRN3* activation also promotes *VRN1* expression, forming a positive feedback loop in this cold-responsive regulatory cascade [146,148]. *VRN4* genes, encoding MADS-box transcription factors, operate similarly to *VRN1* genes [149]. The *VRN1* gene is crucial for flowering initiation in wheat. The timing of wheat heading/flowering varies based on different combinations of dominant and recessive alleles, particularly *VRN-A1* homeologs. Common wheat carries three homologous copies of the *VRN1* locus: *VRN-A1*, *VRN-B1*, and *VRN-D1*. Winter wheat varieties possess recessive alleles (*Vrn-A1*, *Vrn-B1*, and *Vrn-D1* alleles) at these loci, while spring wheat varieties harbor dominant alleles (*Vrn-A1*, *Vrn-B1*, or *Vrn-D1*) at the *VRN1* locus [82]. Insight into the *VRN1* locus has revealed insertions and deletions within its promoter region, as well as a deletion within intron 1. These genetic alterations result in constant *VRN1* expression in spring wheat varieties, enabling flowering without vernalization requirement [129,150,151,152]. Recent studies have shown that dominant alleles of *VRN-A1* typically exhibit indels predominantly located in the promoter sequence, while dominant alleles of *VRN-B1* and *VRN-D1* mostly display deletions within the first introns [151,152,153]. The *VRN-A1* gene’s promoter region contains conservative regulatory elements such as CArG, VRN, and G boxes, collectively influencing the *VRN1* gene’s functional activity [154]. Among these elements, the 16-base pair sequence known as the ‘VRN box’ (“TTAAAAACCCCTCCCC”) is recognized as the most significant factor in determining the ‘winter-spring’ growth habit of wheat [148]. Transferring wild-mutated *VRN-A1* alleles into the common wheat genome without altering the genetic background presents a formidable challenge. Conventional gene transfer methods are labor-intensive and time-consuming. However, the continually advancing CRISPR/Cas9 genome-editing technology offers an exciting opportunity to precisely modify nucleotide sequences within the gene’s promoter region while leaving the remainder of the genome untouched [155,156]. Ref. [82] utilized CRISPR/Cas9 technology to modify the promoter region of the *VRN-A1* gene in plants belonging to the semi-winter cultivar ‘Chinese Spring’. Their genetic alteration involved introducing an 8-base pair deletion occurring within the region spanning from 125- to 117-base pairs of the *VRN-A1* promoter. This manipulation resulted in a notable reduction in the time required for head emergence, shortening it by approximately 2 to 3 days. This study demonstrates the potential of CRISPR/Cas9 technology to manipulate wheat heading time, by altering the promoter sequence without altering the genetic background. Enhancing both yield and early maturity in wheat genotypes through the deliberate manipulation of wheat phenology is feasible, given the negative relationship between days to flowering and grain yield potential. Thus, the objective of such manipulation should be to breed genotypes with accelerated growth rates and increased biomass accumulation, thereby ultimately boosting their yield potential.

## 9. Agronomic Management Practices for Early-Maturing Varieties

For early-maturing wheat cultivars, agronomic packages and practices resemble those of other varieties, albeit with slight variations across regions and production conditions. Timely sowing is paramount for optimal productivity, typically falling within the range of 20 °C to 23 °C average daily temperatures observed during the first half of November [157]. Seed rate recommendations vary based on factors such as seed size, germination percentage, sowing time, water availability, and crop rotation. In India, common recommendations include a row spacing of 20–22.5 cm with a seed rate of 100 kg/ha (at 38 g/1000 seeds), ideally sown using a ferti-seed drill. Fertilizer application should be tailored to soil test values, with a general dose of 150 kg of N, 60 kg of P_2_O_5_, and 30 kg of K_2_O per hectare. Approximately one-third of the nitrogen and full amounts of phosphorus and potassium are advised during sowing, with the remaining nitrogen applied as urea in two splits before the first and second irrigation [158]. To address zinc deficiency, common in rice–wheat cropping systems, around 25 kg of zinc sulfate is applied [78]. Wheat typically requires four to six irrigations, with the crown root initiation and heading stages being critical for managing moisture stress.

## 10. Evaluating Early-Maturation and Short-Duration Varieties under Unpredictable Environmental Conditions

The Indo-Gangetic Plains of South Asia play a critical role in ensuring food security for approximately 900 million people. However, climate change poses a significant threat, potentially rendering these plains unsuitable for wheat cultivation. Concurrently, the rapid depletion of groundwater tables exacerbates concerns over food security and farmer livelihoods. A resilient crop variety is essential to mitigate output losses under these evolving circumstances, particularly as wheat is highly sensitive to heat stress. Northwestern and Central Indian farmers adapt by sowing wheat immediately after rice, capitalizing on residual moisture from the monsoon season to mature before terminal heat stress sets in. However, by mid-November, residual moisture diminishes, necessitating additional irrigation for uniform germination and establishment. In central India, declining water tables by late January render pumping too costly or impossible. Early sowing utilizing residual moisture not only conserves irrigation but also enhances water productivity when combined with other agronomic practices. Early-maturing cultivars offer added benefits by reducing susceptibility to pre-harvest sprouting, a common issue in cold, wet harvests. Strengthening the leaf canopy to shade the soil surface aids in reducing soil erosion, while early season growth enhances leaf transpiration efficiency, ultimately boosting crop water-use efficiency, biomass, and grain yield. Additionally, increased ground cover during early growth stages improves weed competition. The adoption of early maturity as a crop adaptation strategy in high-temperature stress locations has gained traction, with new breeding efforts focusing on early maturity. CIMMYT wheat germplasm has shown promise in diverse climates, contributing to genetic gains in both optimal and stressed environments. For drought-tolerant varieties, evaluating the heading date and effective tiller number is crucial for improving water-use efficiency. Modifying crop development to synchronize phenological phases with seasonal moisture availability is paramount, with a focus on increasing assimilate transport to developing flowers to raise harvest index and grain output. Short-duration wheat varieties are favored for crop rotation due to their reduced input requirements, including irrigation. Ongoing advancements in breeding efforts aim to address these challenges and enhance wheat resilience in water-limited conditions, exemplified by the release and assessment of the Cereal System Initiative for the South Asia Heat-Tolerant Early Maturity (CSISA-HT-EM) lines in India.

## 11. Challenges and Limitations of Earliness in Wheat

The majority of wheat cultivars found in farmers’ fields are ill-suited for early season planting, typically in the third week of October, owing to mild temperatures. These conditions prompt accelerated wheat development, resulting in reduced biomass accumulation and lower yields. Adapted varieties must demonstrate tolerance to both early- and late-season higher temperatures, as advocated by [13]. Despite the benefits of early planting amidst shifting weather patterns, achieving wheat planting in the third week of October remains a challenge for Punjab farmers. In many regions of northwestern and central India, farmers seek to advance wheat planting to capitalize on residual moisture and evade terminal heat stress in March, as discussed by [26]. However, premature growth under warm early seedling conditions leads to diminished biomass, ear, grain counts, and ultimately yield. This problem primarily stems from the inadequate adaptability of current wheat cultivars to elevated temperatures during their early growth and phenological stages. Notably, historical selective breeding efforts have not prioritized enhancing heat tolerance during the juvenile stage, resulting in a knowledge gap in this domain. Furthermore, early-maturing wheat cultivars tend to exhibit lower protein content due to their shorter vegetative development phase, impacting both the texture and cooking qualities of wheat-based products, as indicated by [55].

Meeting the anticipated demand in the coming decades necessitates significant advancements in genetic yield. Moreover, specialized agricultural regions may facilitate adaptation to varying climates. Breeders face the challenge of enhancing traits such as earliness and short duration in improved varieties, requiring three essential components: access to donor germplasm containing favorable alleles for the target trait, methods for trait identification through either phenotypic selection or marker-assisted selection, and adequate human and capital resources to execute selection and breeding strategies.

Enhancing wheat production sustainability can be achieved through reduced water pumping practices. Ref. [159] suggested that the utilization of machinery such as the Happy Seeder, super seeder, and super straw management system in combination facilitates early planting immediately after the harvest process, thereby reducing straw combustion and greenhouse gas emissions. Previously, the lack of suitable early-sowing varieties hindered rapid planting after rice harvest, which typically occurs in October, accounting for approximately 90% of cases. The anticipated introduction of wheat cultivars with early heat tolerance is expected to offer comprehensive benefits for both farmers and environmental sustainability.

Temperature and humidity significantly influence wheat disease, with Fusarium head blight (FHB) serving as a prominent example. Anther retention (AR), while morphologically neutral, is genetically linked to FHB resistance [160]. Warm, damp conditions during anthesis exacerbate FHB severity, rendering wheat heads particularly susceptible to infection during this phase [161], although infection can extend up to the soft dough stage [160]. The introduction of new early varieties may disrupt the wheat disease and insect–pest system, impacting both wheat production and quality. The cyclical nature of wheat rust disease outbreaks underscores the importance of breeding efforts to adapt to evolving pest dynamics. Stem rust, which historically caused significant yield losses, was effectively controlled by resistant wheat varieties until the emergence of the virulent Ug99 lineage in Uganda in 1998 [162]. Additionally, stripe rust has spread to warmer, drier regions, resulting in further crop losses. Local abiotic climate changes affecting wheat yield have the potential to disrupt wildlife habitats and pose environmental risks. The current challenge lies in addressing the rapid pace of pathogen and pest adaptations. Manure and nitrogen fertilizer applications contribute to the emission of N_2_O, a potent greenhouse gas, particularly in intensive wheat-cropping systems where substantial N_2_O and NO fluxes are observed due to fertilizer use [163]. The escalating use of fertilizers and water per crop may lead to the increased pollution of soil and water tables. Such degradation of soils and depletion of water resources pose a threat to the livelihoods of millions of rice–wheat farmers who depend on these crops for sustenance and income. However, optimizing nitrogen quantities and timing in intensive irrigated systems could potentially reduce emissions by up to 50% without compromising wheat yields. Utilizing a handheld optical sensor capable of computing the normalized differential vegetative index (NDVI) to assess yield potential as plants grow can help in minimizing unnecessary nitrogen fertilizer inputs and consequent trace gas emissions, thereby benefiting farmers economically and promoting environmental sustainability [164]. Increasing the number of crops per year, depending on the farm, can have several positive effects, such as reducing soil erosion by minimizing bare land between cropping systems, enhancing soil quality, suppressing weeds, providing habitat for wildlife, and more recently, sequestering carbon. Additionally, early varieties of crops may possess certain desirable characteristics, such as a soft dough consistency, which could be utilized in the production of high-quality agricultural goods, thereby potentially increasing financial gains for farmers.

### 11.1. Successful Implementations of Early-Maturation and Short-Duration Wheat

The farming community views early-maturation or short-duration wheat as highly desirable, as it provides flexibility in crop selection and allows for adjustments in crop cycles on a given arable land. This not only facilitates an increase in the number of crops cultivated annually on a piece of land but also helps in adapting to changing climate conditions [165]. The changing climate scenarios, particularly the occurrence of terminal heat and consistently rising temperatures towards the end of February or early March, pose significant challenges, especially in South Asia. To address this issue, one recommendation is to utilize early-maturing genotypes to mitigate terminal heat stress, as late sowing conditions often lead to reduced crop yields due to decreased kernel weight, particularly in areas where the wheat–rice cropping system is prevalent [24,166].

### 11.2. Early-Maturation and Short-Duration Wheat and Expression of Important Diseases

Earliness in wheat holds significant importance not only in mitigating terminal heat stress and rising temperatures but also as a potential strategy for disease avoidance against various prevalent wheat diseases. Several diseases, such as brown rust (caused by *Puccinia triticina*), stem rust (caused by *Puccinia graminis tritici*), spot blotch (caused by *Bipolaris sorokiniana*), and Fusarium Head Scab disease (primarily caused by *Fusarium graminearum*), thrive in warm temperatures and typically occur later in the crop season. Early-maturing cultivars have the advantage of reaching full biological yield before the onset of these diseases. In case infections do occur, crop losses are generally kept below threshold levels.

## 12. Case Study

In South Asian conditions, the evaluation of wheat genotype adapted to terminal and continual high-temperature stress was conducted by [85], highlighting the emerging challenge of elevated temperatures before crop maturity in the region. The study assessed the grain yield performance of 28 CIMMYT wheat lines, and two checks were selected for their adaptation to high-temperature stress in South Asia. These lines were characterized by their high yield potential, early maturation, and heat resistance. The experiment was carried out across 15 locations, including 13 in South Asia and 2 in Mexico. Each location was categorized into Mega Environments (MEs), with ME1 representing temperate irrigated areas under high-temperature stress and ME5 representing warm, tropical, irrigated locations. Parameters such as grain yield (GY), 1000 kernel weight (TKW), days to heading (DH), plant height (PH), and canopy temperature (CT) were recorded and compared across the locations, revealing significant differences. Cooler ME1 locations exhibited higher mean GY (5.26 t/ha) and TKW (41.8 g) compared to ME5 (3.63 t/ha and 37.4 g, respectively). Entries with early heading (<79 days, mean DH) demonstrated superior performance across all locations, with GY ranging from 2 to 11% above local checks and TKW ranging from 40 to 44 g. The researchers concluded that early-maturing, high-yielding and heat-tolerant wheat lines have the potential to adapt to various heat-stressed areas in South Asia.

## 13. Future Prospects

As global agriculture confronts mounting challenges from climate change, heat stress, and resource constraints, the future of wheat cultivation relies on innovative strategies to navigate these complexities. A pivotal aspect of this endeavor is the development of early-maturing wheat varieties endowed with enhanced heat tolerance, efficient water utilization, and improved nutrient absorption. Employing advanced breeding techniques, such as marker-assisted selection and quantitative trait locus mapping, is essential for identifying and selecting stress-tolerant genotypes.

A significant research avenue involves unraveling the intricate genetic and molecular mechanisms governing nutrient uptake, transport, and their influence on crop productivity in early-maturing wheat genotypes. A deeper comprehension of the genetic architecture of nutrient-related traits can convey targeted breeding strategies aimed at bolstering nutrient use efficiency. Investigating the interplay between environmental factors and hormonal regulation, particularly concerning early maturity, offers promise for cultivating resilient wheat varieties. Integrating advanced omics technologies and precision breeding approaches is crucial for accelerating progress in developing early-maturing wheat genotypes with enhanced nutritional quality and adaptability to shifting climatic conditions. The future of plant breeding is anticipated to witness an integration of advanced technologies and an expanded understanding of the genetic underpinnings of complex traits. Optimizing genomic selection models for more precise and rapid trait enhancement stands out as a key research area. Further exploration of marker-assisted selection and quantitative trait locus mapping, especially for traits associated with climate resilience, will enhance breeding efficiency. The refinement of field phenomics, facilitated by high-throughput phenotyping and novel sensors, will be pivotal for precise trait evaluation across diverse environments. Speed breeding, coupled with modern techniques like genome editing, is poised to play a crucial role in expediting the development of climate-smart crop varieties. The continual refinement of these technologies and their integration into breeding programs will effectively address global food security challenges. However, urgent attention is needed to fill existing gaps in our understanding of the intricate genetic and molecular networks controlling traits such as early maturity and nutrient uptake. In the context of early-maturing wheat, focused research is required to advance genetic modification techniques. Utilizing CRISPR/Cas9 technology holds promise for precisely manipulating key genes like *VRN1* to accelerate flowering without altering the overall genetic background. Additionally, efforts should be directed towards developing wheat varieties with increased heat tolerance during early growth stages, recognizing the gaps in our current understanding of the genetic basis of heat stress resilience. Agronomic practices for early-maturing varieties necessitate further refinement, considering the diverse environmental conditions in which wheat is cultivated. Evaluating the performance of early-maturing varieties under unpredictable climates and addressing challenges related to temperature, disease resistance, and nitrogen management are crucial research areas. The development and implementation of holistic approaches that combine genetic advancements, agronomic practices, and climate-resilient traits are vital for ensuring sustainable wheat production amidst evolving environmental challenges.

In summary, the future of wheat cultivation demands a comprehensive approach that integrates cutting-edge technologies, advances our understanding of genetic mechanisms, and addresses critical knowledge gaps. By prioritizing early-maturing wheat varieties, researchers can contribute to global food security by developing crops resilient to climate change and resource constraints.

## 14. Conclusions

Earliness ensures optimal crop harvest, in addition to protecting wheat from biotic and abiotic stresses such as disease, heat, and dehydration. By accelerating or delaying ear initiation, the vernalization requirement and photoperiod sensitivity safeguard delicate floral primordia from extreme temperatures. This analysis of physiological aspects associated with wheat growth and water use in regions with limited water availability has brought to light certain characteristics that are anticipated to increase water-use efficiency and, consequently, wheat yield. Early flowering and maturity have been identified as a viable mechanism for increasing drought resistance. It is essential to note, however, that this mechanism can potentially limit the potential cereal yield. This limitation arises from the diminished timeframe available for both photosynthetic production and the accumulation of nutrients in the seeds, both of which are crucial for attaining higher cereal yield. Nevertheless, it has been demonstrated that both superficial and deep root systems have a significant capacity for high crop productivity, suggesting that these plants can adapt to drought conditions. This adaptability is enhanced by their capacity to bloom early in their life cycle. Changeable climatic conditions include the occurrence of severe heat and the persistent rise in temperature stress, which are frequently observed in late February or early March, particularly in the South Asian region. In this context, one of the recommendations is the use of early-maturing genotypes to mitigate the effects of terminal heat stress, as late sowing conditions have been observed to reduce crop yields, primarily due to a decrease in kernel weight in areas where a wheat–rice cropping system is preferred.

## Figures and Tables

**Table 1:** Quantitative trait loci (QTLs) associated with traits influencing earliness in wheat.

Trait	QTL Name	Chromosome Location of QTL	References
Days to heading/ Days to flowering	*QDH-2B*	2B	[97]
	*QDH-5A.1*	5A	
	*QDH-5A.2*	5A	
	*QDH-5B*	5B	
	*QDH-7D*	7D	
	*QDH-5A.3*	5A	
	*QFlt.dms-2D.2*	2D	[98]
	*QFlt. dms-5B*	5B	
	*QFlt.dms-2D*	2D	
	*QFlt.dms-7A*	7A	
	*QFlt.dms-6B.2*	6B	
	*QEps.dms-5B1*	5B	[18]
	*wPt-741686*	7A	[99]
	*VRN-D1*	5D	
Days to maturity	*QMat.dms-2D*	2D	[18]
	*QMat.dms-7A.2*	7A	
	*QMat.dms-4A.1*	4A	
	*QEps.dms-5B1*	5B	[18]
	*QEps.dms-1B1*	1B	[18]
	*wPt-741686*	7A	[99]
Flowering time	*QFt.dms-4A1*	4A	[18]
Flag leaf area	*gFla-1B.2*	1B	[95]
	*QFLA-5A.1*	5A	[92]
Tiller numbers	*QTn.ipk-5D*	5D	[89]
	*QTn.ipk-2D*	2D	
	*QTn.ipk-3B*	3B	
	*QTn.ipk-1B*	1B	
Biomass	qPBio-6B2	6B	[100]
Cooler canopy	??	1B, 5A	[27]
	QCtdh.tam-3B	3B	[101]
	*4A-wmc048d*	4 A	[102]
	*C29P13*	7D	[102]
	*6A-gwm617b*	7D	
Photoperiod sensitivity (PS)	4 QTLs	2B, 2D, 5A and 7D	[103]
PS/EPS	*Ppd-D1* region	2D	[104]
PS	*Ppd-D1* region	5B	
EPS	*Ppd-D1* region	6B	
Vernalization requirement (VR)	5QTLs	2B, 5A, 5B, 5D and 6 D	[103]
	*Vrn-A1*	5A	[104]
Intrinsic earliness (IE)	4 QTLs	2B, 2D, 5B and 7A	[103]
Heading Date (HD)/IE	*MQTL1*	2B	[105]
FT/PS/IE	*MQTL2*	2B	
VR	*MQTL3*	2B	
HD/IE	*MQTL4*	2D	
HD/PS	*MQTL5*	2D	
HD/IE	*MQTL6*	2D	
HD/PS	*MQTL7*	4A	
HD/PS	*MQTL8*	4B	
HD/PS	*MQTL9*	5A	
VR/HD	*MQTL10*	5A	
HD/IE	*MQTL11*	5B	
IE/HD-VR	*MQTL12*	5B	
VR/HD-PS	*MQTL13*	5B	
VR	*MQTL14*	5D	
PS/Wh	*MQTL15*	6A	
HD/IE	*MQTL16*	7A	
HD/IE	*MQTL17*	7B	
HD	*MQTL18*	7D	
Earliness per se (*Eps*)	*QEps.dms-1A*	1A	[106]
	*QEps.dms-4A*	4A	
Earliness per se	*Eps-3A^m^*	3A	[107]
	*Eps-3DL*	3D	[98]
	*Eps-1D*	1D	[88]

## Data Availability

No new data were created or analyzed in this study. Data sharing is not applicable to this article.
